# Multilayered Epigenetic Analysis Identifies a Molecular Portrait for Psychological Resilience in Patients With Breast Cancer

**DOI:** 10.1016/j.bpsgos.2025.100545

**Published:** 2025-06-03

**Authors:** Corinna Richter, Olga Dethlefsen, Ulrika Axelsson, Kristina Lundberg, Lisa Rydén, Per Johnsson, Ulrika Ringdahl, Ingalill Rahm Hallberg, Carl A.K. Borrebaeck

**Affiliations:** a CREATE Health Translational Cancer Center, Department of Immunotechnology, Lund University, Lund, Sweden; b National Bioinformatics Infrastructure, Science for Life Laboratory, Stockholm University, Stockholm, Sweden; c Department of Clinical Sciences, Lund University, Lund, Sweden; d Department of Surgery and Gastroenterology, Skåne University Hospital, Malmö, Sweden; e Department of Psychology, Lund University, Lund, Sweden; f Department of Health Sciences, Lund University, Lund, Sweden

**Keywords:** Bioinformatics, Biomarker, Breast cancer, DNA methylation, Epigenetics, Psychological resilience

## Abstract

**Background:**

Psychological resilience refers to a person’s positive adaptation when faced with adversities, such as a breast cancer (BC) diagnosis. Highly resilient patients are more likely to regain stability and be protected from health conditions such as depression, anxiety, and posttraumatic stress disorder. We aimed to identify epigenetic markers that distinguish high- and low-resilient patients in a BC cohort at time of diagnosis.

**Methods:**

Genome-wide DNA methylation was determined in participants selected from a prospectively collected cohort of 1040 newly diagnosed BC patients with known resilience status. DNA methylation of those displaying the highest and lowest scores (*n* = 425), as measured by the Connor-Davidson Resilience Scale, was analyzed in whole blood, using a multilayered bioinformatic approach. Sample subsets were created to identify differentially methylated probes (DMPs) and differentially methylated regions (DMRs), and fold change and area size were used to estimate the strength of methylation differences. The key regions associated with psychological resilience allowed us to build a classifier, using a random forest model, which was validated using an independent cohort (*n* = 80).

**Results:**

DMPs and DMRs that consistently distinguished samples derived from high- and low-resilient patients were identified, and methylation differences followed a dose-response pattern related to resilience levels. DMRs included *LY6G5C, ZFP57, CDH9, ZNF727*, and *C8orf31*, where *LY6G5C* was found to be the most consistent DMR. Psychological resilience status could be predicted in the independent cohort with an area under the curve of 0.74 and a sensitivity and specificity of 0.67 and 0.72, respectively.

**Conclusions:**

*LY6G5C* was identified as a novel marker for psychological resilience, paving the way for a more conceptual and comprehensive molecular understanding.

## Plain Language Summary

Psychological resilience is a personal characteristic that helps preserve stability and QoL when confronted with trauma such as breast cancer (BC) and where highly resilient patients show improved disease outcome. The study explored how DNA methylation was linked to psychological resilience in 425 patients with BC. Key differences were found in methylation patterns, especially in the *LY6G5C* gene, between high- and low-resilient individuals, and a model was built to accurately predict resilience. Findings suggest that *LY6G5C* could be a key biological marker, providing new insights into the association of genetics with psychological resilience and ultimately helping the development of treatments to amend detrimental outcomes.

Psychological resilience refers to the ability for positive adaptation or to maintain or retain mental health despite experiencing adversities (1), such as being diagnosed with breast cancer (BC). Individuals with high scores on the Connor-Davidson Resilience Scale (CD-RISC) demonstrate higher quality of life, especially on mental health factors, than those with lower resilience scores (2, 3, 4), supporting the assumption that they maintain a mental equilibrium despite the unknown ahead. Resilience may protect an individual from stress reactions, such as fear, fatigue or low energy, anxiety, pain, and sleeping problems, or from developing posttraumatic stress disorder (PTSD) or depression (5, 6, 7). Systematic reviews on the prevalence of PTSD (6) and depression (5) in BC have shown these conditions to be common compared with the general population. However, the prevalence varied, for example depending on when in the treatment trajectory PTSD or depression was assessed (5,6). Furthermore, research has indicated that resilience protects against developing PTSD or depression (8) when confronted with a traumatic event such as BC. Consequently, there has been increasing interest in the concept of resilience in cancer research, not only because highly resilient cancer patients are less likely to suffer from mental health complications but also because they experience better treatment outcomes (2,3,7).

Although there is strong evidence that psychological resilience plays an important role in how people manage and adapt to challenging life situations, including health threats, there is no consensus yet about whether psychological resilience is a personal characteristic or a dynamic process and only limited knowledge about how it is related to biological aspects, such as neurochemical and (epi)genetic factors (9). DNA methylation (DNAm) is one of the best-characterized epigenetic mechanisms and has been shown to be involved in regulating many cellular functions. Epigenetic modifications have also received particular attention because they can, in contrast to the genetic code, change in response to environmental factors (10). Thus, epigenetic markers of psychological resilience could help identify vulnerable individuals, inform about the impact of traumatic events, and/or monitor the success of therapeutic interventions. For several psychiatric disorders, the importance of different genes and their methylation status is well established. Many DNAm studies target genes with a connection to brain function or stress response, such as *BDNF* (11), *NR3C1* (12), *FKBP5* (13), or *MAOA* (14). The DNAm status of these candidates have regularly been associated with multiple psychiatric conditions (15, 16, 17, 18). Epigenome-wide association studies have successfully identified unexpected candidates, such as *AHRR*, that emerged as epigenetic loci associated with PTSD (19,20).

Here, we used samples from the SCAN-B (Swedish Cancerome Analysis Network-Breast) Resilience study (4,21), the largest prospectively collected cohort of newly diagnosed BC patients with known resilience scores to date, to define an epigenetic portrait of psychological resilience. We identified single CpGs as well as genomic regions for which methylation patterns differed between participants with high and low psychological resilience. Furthermore, we demonstrated that this difference in methylation signal displayed a dose-response–like configuration and that CpGs located in the *LY6G5C* genomic region dominated in a random forest classifier trained and validated to discriminate between BC patients with high and low resilience.

## Methods and Materials

### Study Design

The current study, denoted SCAN-B Resilience (clinical trial ID: NCT03430492), is an amendment to the SCAN-B initiative and is a multicenter study conducted at 4 hospitals in southern Sweden during 2016 to 2019. The protocol was published (21) and the study was approved by the Regional Ethical Review Board, Lund University, and the Swedish Ethical Review Authority. At the time of consultation for BC diagnosis, participants provided a blood sample, and their psychological resilience was measured using the Swedish version of the 25-item resilience scale by Connor and Davidson (CD-RISC) (22). This self-report measure consists of 25 statements rated on a 5-point Likert scale. Scores range from 0 to 100, with higher scores representing higher resilience. Factor structure exploration suggested 5 factors assessing 1) personal competence, high standards, tenacity; 2) tolerance of negative affect and strengthening effects of stress; 3) positive acceptance of change and secure relationships; 4) control; and 5) spiritual influence. These factors have been shown to be unstable in other research, and thus the authors recommend using only a summed score. The CD-RISC has shown to have good psychometric properties (22) and to be sensitive to treatment (22). Mean imputation was performed for up to 6 missing items, as recommended to ensure validity (https://cd-risc.com/faq.php). The validity of the scale in the Swedish context was confirmed prior to this study (23). Clinical variables were obtained from the Swedish National Breast Cancer Register.

### Study Participants

**Figure 1:**
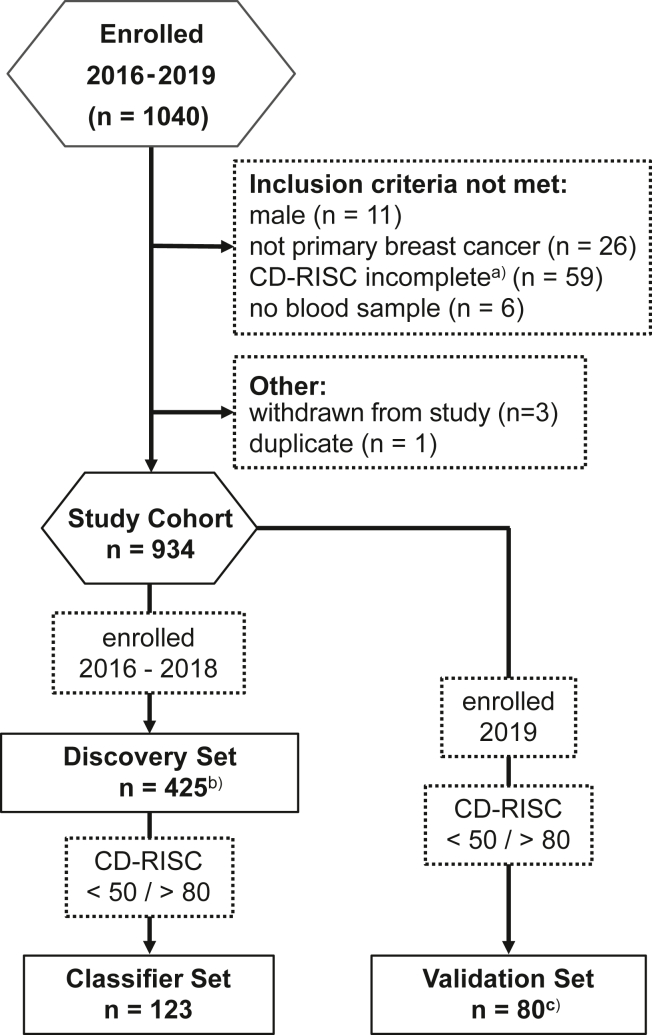
Study outline. Flow of patients and datasets generated for the analysis of the association between methylation patterns and resilience status. ^a^More than 6 of 25 items missing. ^b^Two samples failed QC. ^c^One sample failed QC. CD-RISC, Connor-Davidson Resilience Scale; QC, quality control.

Study participants were enrolled into SCAN-B Resilience between 2016 and 2019 (*N* = 1040) based on informed consent. Inclusion criteria for the epigenetic investigation specified female, newly diagnosed patients with primary BC, age ≥ 18 years, and able to understand and speak the Swedish language. Participants had to provide a 15-mL blood sample and complete the CD-RISC (*N* = 934) (Figure 1, Table 1, and Figure S1). With no defined cutoff scores for low and high resilience available for the CD-RISC, 425 patients with the highest and lowest CD-RISC scores were selected for the discovery set. From the discovery set, 10 sample subsets were then created based on CD-RISC score percentiles, where samples were removed from the middle ranges in steps of 10%; i.e., subset 10% would contain the 10th percentile highest samples of the high-resilient group and the 10th percentile lowest samples of the low-resilient group. The classifier set (*n* = 123) was sourced from the discovery set after the differentially methylated probe/differentially methylated region (DMP/DMR) analysis to train a random forest classifier (Figure 1 and Figure S1) distinguishing low- and high-resilient patients. Here, each sample from low-resilient patients (score ≤ 50) was matched with 2 samples from high-resilient patients (score ≥ 80). To eliminate potential covariate effects, samples were matched based on age, tumor characteristics (progesterone receptor, estrogen receptor [ER], and HER2 status), and smoking. Daily, occasional, and previous smokers were combined into ever smokers, while never smokers where matched only to never-smokers. The validation set (*n* = 80) consisted of independent test samples that had not previously been analyzed and selected from patients enrolled during 2019 (Figure 1, Figure S1, and Table 2).

### DNAm Analysis

DNA was purified from whole blood. DNAm status was assessed using the Infinium MethylationEPIC v1.0 BeadChip (Illumina Inc.). Analyses were performed at the Center for Translational Genomics and Clinical Genomics, Lund University, Lund, Sweden (discovery set) or at Eurofins Genomics A/S, Galten, Denmark (validation set).

### Data Processing, Annotation, and Quality Control

Raw EPIC array methylation files (IDAT) were processed in R (version 4.2.1), using the ChAMP pipeline (version 2.21.1) (24). Briefly, probes were filtered based on 1) *p* values > .01; 2) the presence of at least 3 beads in at least 5% of samples per probe; and exclusion of 3) non-CpG probes, 4) single nucleotide polymorphism–related probes, 5) multihit probes, and 6) probes located on the X and Y chromosomes. After quality control (QC) based on density and multidimensional scaling plots, beta values were normalized (25), and singular value decomposition analysis for batch effects was run to assess technical effects of methylation date, array, slide, and plate. Batch effects associated with methylation date were corrected using ComBat (26). Probe genomic coordinates were obtained from the EPIC hg19 manifest. The Annotatr package (version 1.26.0) was used to annotate probes to genomic features, such as CpG annotations (CpG islands, shores, shelves, and open sea) and genetic annotations (e.g., promoters, introns, exons). To eliminate an effect of different cell types as a confounder, cell-type proportions in the samples were estimated using epiDISH (version 2.18.0) (27) and 3 reference-based algorithms: robust partial correlations, robust penalized multivariate regression implementation, and Houseman’s linear constrained projection (28).

### Differential Methylation Analysis

To stabilize variance and approximate normality, β values were transformed to M values using logit transformation. Limma (version 3.58.1) was used to fit a linear model for every CpG site in the discovery set accounting for age, menstrual status, detection mode, and ER status to find CpGs associated with low CD-RISC scores (Table 3 and Figure S1). Next, we attempted detection of resilience-associated methylation patterns that might be obscured in a full-cohort analysis by stratifying the discovery set into 10 subsets using CD-RISC score percentiles (Table 3). CpGs were considered as DMPs between the low- and high-resilient groups at *p* < .05 and log_2_ fold change <−0.5 or >0.5, corresponding to an approximately 10% to 15% difference in methylation on the β value, commonly considered a moderate effect size and allowing for detection of subtle changes (29). DMRs were called using Bumphunter (version 1.44.0) (30) with default settings, accounting for age, menstrual status, detection mode, and ER status. The DMR area is defined as the absolute value of the sum of the estimated model coefficients in the region (30) and was used to estimate the degree of differential methylation. To mitigate false positives, we focused on DMPs and DMRs that were consistently detected across multiple subsets, also incorporating the signal strength (log_2_ fold change). β values were retained for visualization and interpretation.

**Table 3:** Subsets of the Discovery Set

Subset	CD-RISC		*p* Value
	Low-Resilience Group	High-Resilience Group	
All	*n* = 162	*n* = 167	<.001
	55.9 (7.5) [32–65]	83.8 (5.5) [77–100]	
90%	*n* = 156	*n* = 143	<.001
	55.6 (7.4) [32–64]	84.8 (5.2) [79–100]	
80%	*n* = 143	*n* = 120	<.001
	54.8 (7.2) [32–63]	85.9 (5.0) [80–100]	
70%	*n* = 119	*n* = 109	<.001
	53.2 (6.9) [32–61]	86.5 (4.8) [81–100]	
60%	*n* = 99	*n* = 98	<.001
	51.8 (6.7) [32–59]	87.1 (4.7) [82–100]	
50%	*n* = 86	*n* = 71	<.001
	50.7 (6.5) [32–58]	88.9 (4.3) [84–100]	
40%	*n* = 68	*n* = 62	<.001
	48.9 (6.1) [32–56]	89.6 (4.2) [85–100]	
30%	*n* = 52	*n* = 48	<.001
	46.9 (5.7) [32–53]	90.8 (4.1) [87–100]	
20%	*n* = 33	*n* = 29	<.001
	43.9 (5.2) [32–50]	93.0 (3.9) [89–100]	
10%	*n* = 18	*n* = 17	<.001
	40.2 (4.1) [32–45]	95.3 (3.5) [91–100]	
5%	*n* = 10	*n* = 9	<.001
	37.4 (3.3) [32–41]	98.3 (1.6) [96–100]	

CD-RISC values are presented as mean (SD) [range].

CD-RISC, Connor-Davidson Resilience Scale.

### Random Forest

As an additional layer of analysis, we applied a machine learning approach to identify CpGs with predictive value for resilience. The classification set (*n* = 123) was subjected to 500 iterations of stratified random partitioning into training (50%), confirmation (20%), and test (30%) sets, with each set preserving the inherent triplets of 1 low-resilient sample matched with 2 high-resilient samples. In each iteration, the relevant features (CpGs) were identified as those located in DMRs that spanned over at least 5 CpG probes and distinguished between low- and high-resilient samples. The confirmation set was used to further filter for relevant CpGs found in the DMRs by retaining only probes consistently hyper- or hypomethylated in both the training and confirmation sets (based on fold-change comparison). Ranger implementation (version 0.16.0) (31) of the random forest algorithm was used to train a classifier that distinguished low- and high-resilient samples using the relevant CpGs as features and 70% of the samples (including both the training and confirmation sets). Model parameters (mtry, number of trees, minimum node size, maximum depth, splitrule) and classification threshold were tuned, using nested k-fold cross-validation with 5 folds (k = 5) and 3 repeats (m = 3), maximizing Youden’s *J* statistics (*J* = sensitivity + specificity − 1) to account for imbalanced data. The tuned model was finally assessed on the test set. Across the 500 iterations, CpGs were ranked based on the frequency of appearance. The final random forest classifier was tuned using the entire classifier set (*n* = 123), again tuning model parameters and classification threshold using nested k-cross validation (k = 5, m = 3). The model’s performance was evaluated on the validation set (*n* = 79), which comprised independent samples.

### Statistical Analyses

Assessment of differences in group demographics and clinical variables was carried out in R using the gtsummary package (version 1.7.0). *p* Values were obtained using Pearson’s χ^2^ square tests for categorical variables with all expected cell counts >5 and Fisher’s exact tests for categorical variables with any expected cell count <5. For continuous variables, Wilcoxon rank-sum tests were used. Unless stated otherwise, *p* < .05 was considered statistically significant.

## Results

### Study Design and Cohort Demographics

The study design and flow of participants are illustrated in Figure 1. Demographics, clinical variables, and CD-RISC distributions of the study cohort and the sample sets are summarized in Tables 1 and 2 and Figure S1. In the discovery set, high-resilient (*n* = 212) and low-resilient (*n* = 213) participants differed in age (*p* = .028) and menstrual status (*p* = .004), with high-resilient patients being somewhat younger. The only cancer-related variable that differed between high- and low-resilient participants was ER status (*p* = .038) (Table 1).

**Table 1:** Demographic and Clinical Variables of the Study Cohort and Discovery Set

	Study Cohort, *N* = 934	Discovery Set		
		High, *n* = 212	Low, *n* = 213	*p* Value^a^
CD-RISC Score	71 (13)	84 (5)	56 (8)	<.001
Age, Years	62 (11)	61 (12)	64 (11)	.028
Menstrual Status				
Postmenopausal	660 (80%)	147 (75%)	167 (87%)	.004
Premenopausal	161 (20%)	49 (25%)	26 (13%)	
Unknown	113	16	20	
Cancer Detection Mode				
Screening	547 (61%)	117 (57%)	129 (63%)	.28
Symptomatic	354 (39%)	87 (43%)	77 (37%)	
Unknown	33	8	7	
Cancer Stage				
0	46 (5.1%)	9 (4.4%)	12 (5.9%)	.41
I	564 (63%)	121 (60%)	131 (65%)	
II	266 (30%)	68 (33%)	58 (29%)	
III	15 (1.7%)	5 (2.5%)	2 (1.0%)	
IV	6 (0.7%)	–	–	
Unknown	37	9	10	
Histology				
Ductal	620 (79%)	136 (75%)	146 (80%)	.16
Lobular	93 (12%)	31 (17%)	19 (10%)	
Mixed	76 (9.6%)	14 (7.7%)	18 (9.8%)	
Unknown	145	31	30	
ER Status				
Negative	106 (14%)	33 (18%)	19 (11%)	.038
Positive	677 (86%)	146 (82%)	159 (89%)	
Unknown	151	33	35	
PR Status				
Negative	251 (32%)	63 (35%)	51 (29%)	.18
Positive	531 (68%)	116 (65%)	127 (71%)	
Unknown	152	33	35	
HER2 Status				
Negative	689 (89%)	155 (87%)	159 (89%)	.61
Positive	88 (11%)	23 (13%)	20 (11%)	
Unknown	157	34	34	
Smoking				
Ever	93 (13%)	26 (15%)	18 (10%)	.23
Never	640 (87%)	152 (85%)	155 (90%)	
Unknown	201	34	40	

Values are presented as mean (SD), *n* (%), or *n*. For smoking, ever includes daily, occasional, and previous smokers.

CD-RISC, Connor-Davidson Resilience Scale (25 items); ER, estrogen receptor; PR, progesterone receptor.

a Wilcoxon rank-sum test, Pearson’s χ^2^ test, Fisher’s exact test.

**Table 2:** Demographics and Clinical Variables of the Classifier and Validation Sets

	Classifier Set			Validation Set		
	High, *n* = 82	Low, *n* = 41	*p* Value^a^	High, *n* = 68	Low, *n* = 12	*p* Value^a^
CD-RISC Score	86 (5)	44 (6)	<.001	87 (5)	46 (4)	<.001
Age, Years	65 (10)	65 (10)	.88	62 (11)	65 (10)	.25
Menstrual Status						
Postmenopausal	68 (88%)	33 (92%)	.75	42 (78%)	8 (80%)	>.99
Premenopausal	9 (12%)	3 (8.3%)		12 (22%)	2 (20%)	
Unknown	5	5		14	2	
Cancer Detection Mode						
Screening	40 (50%)	26 (67%)	.086	40 (62%)	8 (73%)	.74
Symptomatic	40 (50%)	13 (33%)		25 (38%)	3 (27%)	
Unknown	2	2		3	1	
Cancer Stage						
0	3 (3.8%)	1 (2.6%)	.71	7 (11%)	0 (0%)	.66
I	49 (62%)	28 (74%)		41 (63%)	7 (64%)	
II	26 (33%)	9 (24%)		16 (25%)	4 (36%)	
III	1 (1.3%)	0 (0%)		1 (1.5%)	0 (0%)	
Unknown	3	3		3	1	
Histology						
Ductal	56 (78%)	25 (71%)	.50	46 (84%)	7 (78%)	.81
Lobular	11 (15%)	5 (14%)		5 (9.1%)	1 (11%)	
Mixed	5 (6.9%)	5 (14%)		4 (7.3%)	1 (11%)	
Unknown	10	6		13	3	
ER Status						
Negative	8 (11%)	6 (17%)	.54	7 (12%)	1 (11%)	>.99
Positive	62 (89%)	29 (83%)		50 (88%)	8 (89%)	
Unknown	12	6		11	3	
PR Status						
Negative	23 (33%)	13 (37%)	.66	15 (27%)	4 (44%)	.43
Positive	47 (67%)	22 (63%)		41 (73%)	5 (56%)	
Unknown	12	6		12	3	
HER2 Status						
Negative	64 (91%)	33 (94%)	.72	52 (95%)	8 (89%)	.46
Positive	6 (8.6%)	2 (5.7%)		3 (5.5%)	1 (11%)	
Unknown	12	6		13	3	
Smoking						
Ever	12 (17%)	4 (12%)	.51	3 (6.7%)	2 (22%)	.19
Never	60 (83%)	30 (88%)		42 (93%)	7 (78%)	
Unknown	10	7		23	3	

Values are presented as mean (SD), *n* (%), or *n*. For smoking, ever includes daily, occasional, and previous smokers.

CD-RISC, Connor-Davidson Resilience Scale (25 items); ER, estrogen receptor; PR, progesterone receptor.

a Wilcoxon rank-sum test, Pearson’s χ^2^ test, Fisher’s exact test.

### Quality Control and Overall Data Structure

During QC assessment of the DNAm data, a total of 714,959 CpG probes (approximately 84%) and 423 samples of the discovery set passed. In peripheral blood, variation in cell composition across different samples could confound associations of DNAm with modeled outcomes (28), and therefore we estimated cell-type compositions using 3 different algorithms. However, no differences in cell-type composition between the high- and low-resilient samples were detected irrespective of the estimation methods used (Figure S2).

Psychological resilience is a complex trait, and the methylation signal could be affected by other variables and confounding factors. Consequently, prior to investigating resilience-associated differential methylation, we assessed the overall data structure by principal component analysis (PCA) of the discovery set (*n* = 423), using the 714,959 CpG probes that had passed QC. Formation of 2 clusters was observed, where PC1 and PC2 captured the most variance, with 13.51% and 10.57%, respectively. However, neither any available clinical variables nor resilience were associated with the first 2 PCs (Figure S3). PC3 (6.48% of the variance) was significantly associated (*p* < .05) with age, menstrual status, and smoking (Figure S3 and Table S1), whereas PC4 (2.02% of the variance) was significantly associated with resilience (Table S1). Thus, while resilience explained some of the variance in the data, the largest source of variation was not explained by known factors, underlining the importance of an extra level of data control to avoid potential confounding factors influencing the outcome.

### Psychological Resilience–Associated Differential Methylation

**Figure 2:**
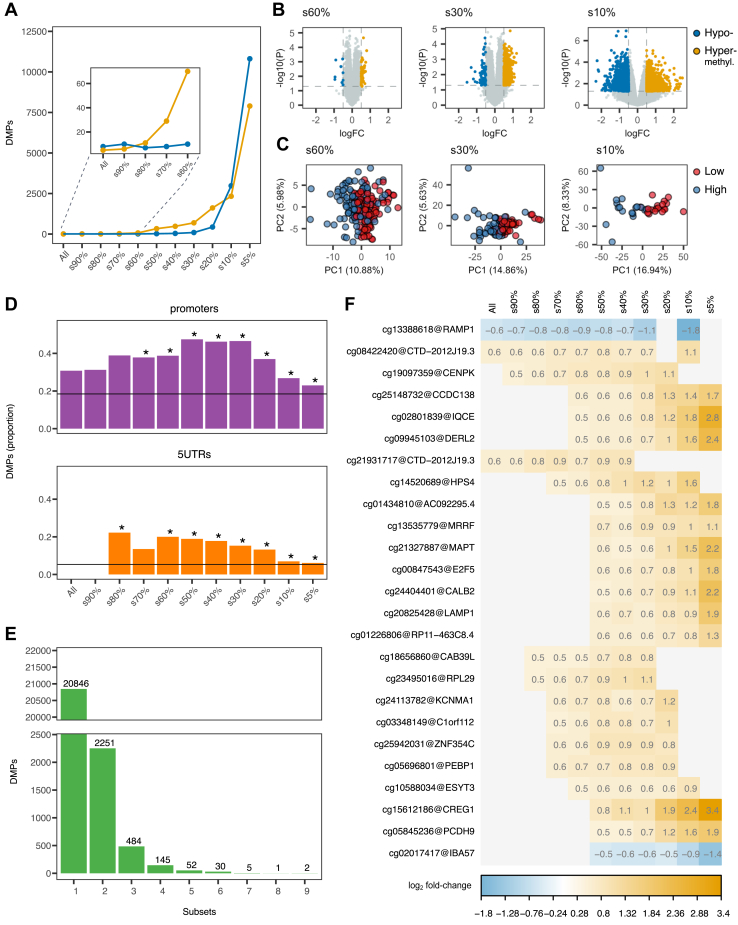
Association of methylation levels at individual CpG sites and resilience status. **(A)** Number of CpGs identified as DMPs between high- and low-resilient samples when comparing all samples in the discovery set and across its 10 subsets. **(B)** Volcano plots showing the statistical significance of DMPs relative to the log_2_ FC for every CpG site for 3 selected subsets (60%, 30%, and 10%). **(C)** PC analysis score plots corresponding to **(B)** based on the CpGs identified as DMPs for the subsets mentioned above, with samples color coded by resilience status. **(D)** Proportions of DMPs overlapping with promoter and 5′ untranslated genomic regions, with the horizontal reference line corresponding to the proportions of all CpG probes in the methylation array passing quality control. ∗Proportions significantly increased/decreased (*p* < .05). **(E)** Bar plot showing the number of subsets in which individual CpGs were identified as DMPs. **(F)** Visualization of the strength of association (log_2_ FC) between resilience and methylation for the 25 top DMPs in the discovery set and its 10 subsets. DMP, differentially methylated probe; FC, fold change; PC, principal component; s, subset.

**Figure 3:**
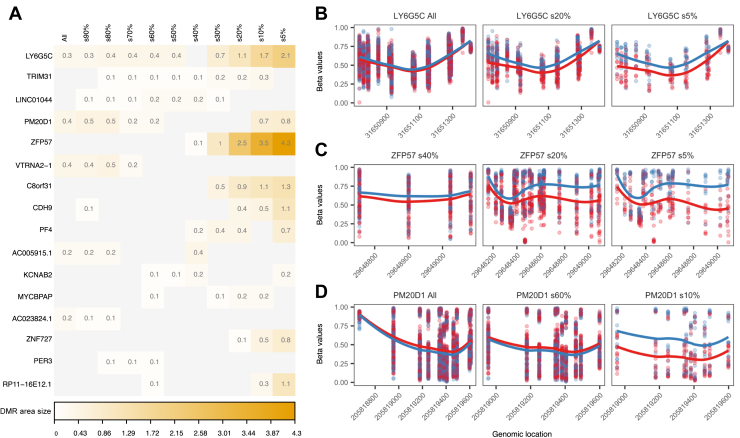
Association between methylation levels at genomic regions and resilience status. **(A)** Visualization of the strength of association (DMR area size) between resilience and methylation for the top DMRs in the discovery set and its 10 subsets. **(B–D)** Examples of DMRs identified when comparing high- and low-resilient samples in the discovery set and representative subsets. Points show methylation measurements in samples obtained from participants with low (red) and high (blue) resilience status. Curves represent the smooth estimate of the methylation profiles in the respective resilience groups. DMR, differentially methylated region; s, subset.

In the linear model, 21,169 CpGs were statistically associated with CD-RISC scores at *p* < .05 before correction for multiple testing. However, as in many EPIC array studies, where standard approaches have been shown to be overly conservative (32,33), none remained significant after adjusting for multiple testing (Table S2). Because it is unknown at which CD-RISC score underlying biological mechanisms would be detectable, i.e., which low or high score would reflect an altered molecular function, we assumed that resilience-associated methylation patterns might be obscured in a full-cohort analysis but may become visible when moving toward the distal ends of the scale. Consequently, we dissected the discovery set (100%) into 10 subsets based on CD-RISC score percentiles (Table 3) and performed differential methylation analysis on each, incorporating log_2_ fold change as a measure of biological relevance. Using *p* < .05, log_2_ fold change ±0.5 as the cutoff, 27,881 DMPs were identified when we compared all high- and low-resilient samples. As hypothesized, the number of DMPs increased toward the distal tails of the resilience scores (Figure 2A), with a distinct rise in hypermethylation in subset 60% and hypomethylation in subset 30%. This resembled a dose-response pattern, because larger differences in CD-RISC scores between groups resulted in larger fold changes (Figure 2B). Improved separation of the high- and low-resilience groups in PCA plots (Figure 2C) confirmed that the differential methylation was attributable to resilience rather than unknown factors. Annotation of the identified DMPs to functional genomic regions revealed overrepresentation in promoters and 5′ untranslated regions (Figure 2D), both regulatory elements for gene transcription, and underrepresentation in exons, introns, or intergenic regions (Figure S4). To identify biologically plausible DMPs, we next analyzed how often an individual CpG was detected as a DMP across all subsets (Figure 3E). The majority of CpGs were identified as DMPs in only 1 (*n* = 20,846, 87.5%) or 2 (*n* = 2251, 9.5%) subsets, confirming the risk of identifying false positives. However, multiple CpGs were identified as DMPs in up to 9 different subsets, with up to 8 being consecutive (Figure 3F). Again, a dose-response–like pattern was observed, with several DMPs exhibiting an association of log_2_ fold change with increasing differences in mean CD-RISC scores, confirming their legitimacy as a marker for resilience. Functions of genes annotated to those CpGs have been associated with basic cell functions such as degradation of misfolded proteins in the ER (*DERL2*), promotion of cell differentiation and autophagy (*CREG1*), involvement in neural signaling by stabilizing the receptor of neuropeptide CGRP (*RAMP1*), and facilitating induction of hippocampal long-term potentiation (*CALB2*) (34, 35, 36, 37). *E2F5*, *MAPT*, and *PCDH9* were particularly interesting in the resilience context. *E2F5* messenger RNA was recently proposed as a target for 3 anxiolytic microRNAs (38), while plasma tau (MAPT) has shown association with measures of depression in older, cognitively intact adults (39). Low levels of PCDH9 in brain and peripheral blood (40) have been associated with major depressive disorder. Consequently, by dividing the discovery set into 10 subsets and performing differential methylation analysis, it was possible to narrow down the identified DMPs to those representing CpGs associated with psychological resilience.

DMRs, which are genomic areas that comprise several DMPs, are more likely to be biologically relevant than individual DMPs (33). Analogous to the DMP analysis, we used the 10 subsets of the discovery set to identify DMRs and found a trend comparable to that of the DMPs, with larger numbers of DMRs in the distal subsets (Figure S5). Because fold changes are not applicable to DMRs, we used the DMR area (30) as surrogate to indicate the strength of identified differences in the methylation profiles. Similarly to the DMP analysis, larger area sizes of >0.5 occurred in the more distal subsets (Figure 3A). When we assessed occurrence and area size for individual DMRs, some did not exhibit an increase in area size or exhibited an irregular nonconsecutive pattern, demonstrating that methylation status was not linked to psychological resilience. This included DMR *PM20D1*, and although it has been suggested that methylation levels of this region are associated with PTSD, this finding was not reproducible (41). Other DMRs combined presence in consecutive subsets with increasing area size, confirming their association with resilience. Among these identified gene regions, we found *C8orf31*, *CDH9*, and *ZNF727*, with the latter 2 having been linked to cognition (42,43). DMR *LY6G5C* was of particular interest because this region was detected in all except one subset, displaying a methylation profile with a distinct dose-response–like trend (Figure 3A, B). On the other hand, DMR *ZFP57* exhibited the strongest increase in region area, consecutive over subsets 40% to 5%. This is notable because hypomethylation of the *ZFP57* region has reproducibly been associated with occurrence of PTSD symptoms, and a follow-up study showed that hypomethylation was reversed in individuals who responded to PTSD therapy (41,44). In concordance with this, our data showed that *ZFP57* was hypomethylated in low-resilient participants (Figure 3C). Taken together, our strategy has allowed us to identify relevant epigenetic loci and identified *LY6G5C* as a strong novel candidate for a molecular marker of psychological resilience.

### A *LY6G5C*-Based Classifier for High and Low Psychological Resilience

**Figure 4:**
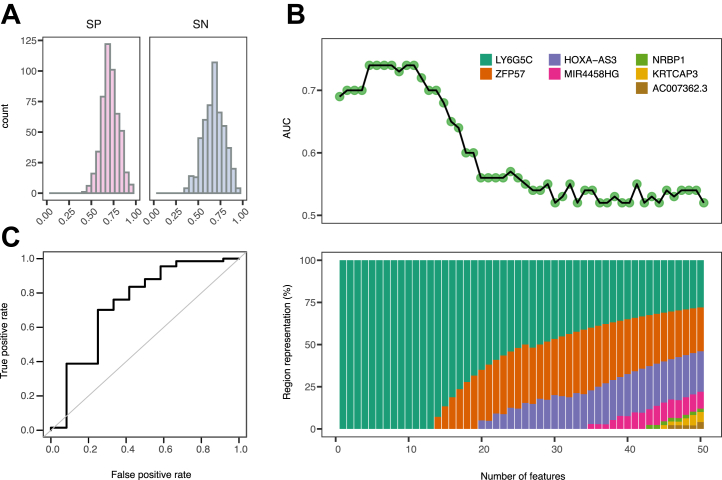
Random forest classifier for distinguishing samples from high- and low-resilient patients based on methylation measurements. **(A)** Distribution of SP and SN obtained when testing the random forest classifier across 500 runs of splitting the classifier set into train, confirmation, and test sets. **(B)** AUC as a function of number of features (CpGs) used to construct the random forest model (top) and visualization of the genomic annotations of the features used (bottom) showing the proportions of different differentially methylated regions contributing to the classifier. **(C)** A receiver operating characteristic curve illustrating the performance of the final random forest classifier assessed on the validation set. AUC, area under the curve; SN, sensitivity; SP, specificity.

Based on our findings of several regions likely to be associated with psychological resilience (*LY6G5C*, *ZFP57*), we built a random forest classification model to predict psychological resilience status. We used our classifier set (*n* = 123), in which we matched each sample with a CD-RISC score <50 with 2 samples with a CD-RISC score >80 (Table 2). Due to the discovered irregularities mentioned above in, e.g., DMR *PM20D1* (Figure 3A, D), we added a further filtering step limiting training to CpGs in regions identified as DMRs for which the direction of fold change remained the same in the confirmatory set. To construct the final model, we first aggregated results across 500 models trained by randomly repeating the data splitting. A total of 2142 CpGs contributed to the models across the 500 runs. Notably, CpGs located in the *LY6G5C* region were the most consistently identified features, occurring in 402 to 474 of 500 runs. Among the 50 most common CpGs, we also detected those in the *ZFP57* region, which was consistent with our previous findings in the discovery set. Across the 500 runs, low-resilient samples were correctly classified with a mean area under the curve (AUC) of 0.70 (±0.19 SD), mean sensitivity (SN) of 0.67 (±0.11 SD), and mean specificity (SP) of 0.71 (±0.10 SD) (Figure 4A).

To build the final model, we used CpGs ranked by their contributions to the 500 models. We noted that the best-performing models in terms of high AUC were obtained when using between 6 and 9 of the top CpGs. Strikingly, the top 9 probes were exclusively annotated to *LY6G5C* (Figure 4C and Figure S6). The final model included 9 features, 8 CpGs, and age, the only clinical variable accounted for due to the well-established link between age and methylation status. We tested the final model in an independent validation set containing samples collected during 2019 (*n* = 79 passed QC) and with CD-RISC scores ≤50 (*n* = 12) or ≥80 (*n* = 67). Classifying these independent validation samples yielded predictions of resilience status with an AUC of 0.74, a SN of 0.67, and a SP of 0.72, thus confirming the potential of the classifier for psychological resilience (Figure 4C).

## Discussion

There is growing evidence that epigenetic mechanisms are involved in an individual’s ability to manage and adapt to traumatic events. Here, we identified epigenetic loci associated with resilience and presented an epigenetic classifier based on the whole-blood methylome, discriminating high- and low-resilient patients with BC.

Our identification of the *LY6G5C* region, which showed the strongest discriminating ability related to psychological resilience, is a novel finding. Although our identified DMPs did not match 3 CpGs (cg18565204/*AARS*, cg17682313/*FBXW7*, cg07167608/*LINC01107*) in a recently defined resilience classifier (45), we identified *ZFP57* as a DMR, the methylation status of which has been repeatedly linked to PTSD (41,44). Even the development of the random forest classifier showed that CpGs from both *ZFP57* and *LY6G5C* regions were among the most consistently identified features, but the best performance of the machine learning model was achieved with 8 CpGs located in the *LY6G5C* region. Importantly, we were able to confirm the discriminatory power of *LY6G5C* in an independent validation dataset. *LY6G5C*, as part of the Ly6 gene family and belonging to the Ly6/uPAR superfamily, is characterized by an LU domain with a specific cysteine pattern creating a 3-fingered structural motif, the exact function of which has not yet been identified in humans (46). There are isoforms predicted to be membrane bound, but experimental analysis suggested that LY6G5C belongs to the secreted members of the Ly6 protein family rather than being GPI anchored (47,48). Fluorescence-activated cell sorting (FACS) analysis of different cell lines has suggested ligands for LY6G5C on undifferentiated megakaryocytes, macrophages, and B cells (47). In blood, *LY6G5C* RNA is expressed across most immune cell compartments with low specificity for a certain cell type (48,49). *LY6G5C* RNA expression is enhanced in brain tissue, particularly in the cerebellum and basal ganglia (48,50). The function of *LY6G5C* is unknown, although it was shown in mice that hippocampal transcription of the *LY6G5C* homolog was downregulated in offspring affected by maternal immune activation (MIA). MIA can have a lasting negative impact on neurodevelopment of the offspring, including hippocampal dysfunction (51). This is relevant because the hippocampus, as part of the limbic system, is involved in memory formation and learning, including appropriate response and the resulting resilience to stress (52). In this context, it is important to recognize that the CpGs that form the *LY6G5C* DMR are not located in the promoter but rather in exons and introns, where methylation has been associated with increased translation rates (53), congruent with our finding that high-resilient individuals show higher methylation of the *LY6G5C* region. In concordance, another study showed that DNAm of *LY6G5C* in peripheral blood mononuclear cells was inversely correlated with cognitive decline (54). While additional studies are required to identify its exact biological role, our study further supports the involvement of *LY6G5C* in maintaining healthy brain function and improved physiological stress response, the latter being a signum for high-resilient participants.

Our investigation has strengths and limitations. Our multilayered analytical strategy was designed to identify biologically plausible methylation patterns that may be missed by traditional testing for significance. Observing consistent direction and effect size across multiple percentile-based stratifications suggests robustness. We also acknowledge important limitations. The stratified subsets are not independent samples, and repeated analysis of overlapping data may increase the risk of detecting internally consistent but nongeneralizable patterns. Without multiple testing correction in the subset analyses, the potential for type I error remains. A general limitation relates to all participants being female, which makes extrapolations to male individuals an issue, and a further generalization of the classifier to predict resilience trajectories over time is not possible. Given its strengths, including interrogation of the largest prospectively collected cohort of representative patient samples and independent validation of the random forest classifier, our study presents an exploratory framework to prioritize candidate loci for further investigation and demonstrates the potential in utilizing epigenomic patterns for diagnostic or even interventional purposes to foster resilience in vulnerable individuals.

### Conclusions

Our study demonstrated that it is possible to identify and validate molecular markers of complex traits, such as psychological resilience, and supports the notion that perceptions and emotions influence bodily functions. We showed that the level of methylation at several CpGs and genomic regions correlated with the degree of psychological resilience. Furthermore, the identification of the novel *LY6G5C* locus allowed us to establish a CpG-based classifier, which clearly marks the somatic association between body and mind regarding psychological resilience.

## Supplementary Material

Table S2

Key Resources Table
