# Supplementary material for: Multilayered Epigenetic Analysis Identifies a Molecular Portrait for Psychological Resilience in Patients With Breast Cancer
Source: Biol Psychiatry Glob Open Sci. 2025 Jun 3;5(5):100545. doi: 10.1016/j.bpsgos.2025.100545 (PMC12281357; doi:10.1016/j.bpsgos.2025.100545)
Supplement: Figures S1–S6 and Table S1 [file mmc1.pdf]

SUPPLEMENTARY INFORMATION

Multi-Layered Epigenetic Analysis Identifies a Molecular Portrait for Psychological Resilience in Breast Cancer Patients

Richter *et al.*

a)

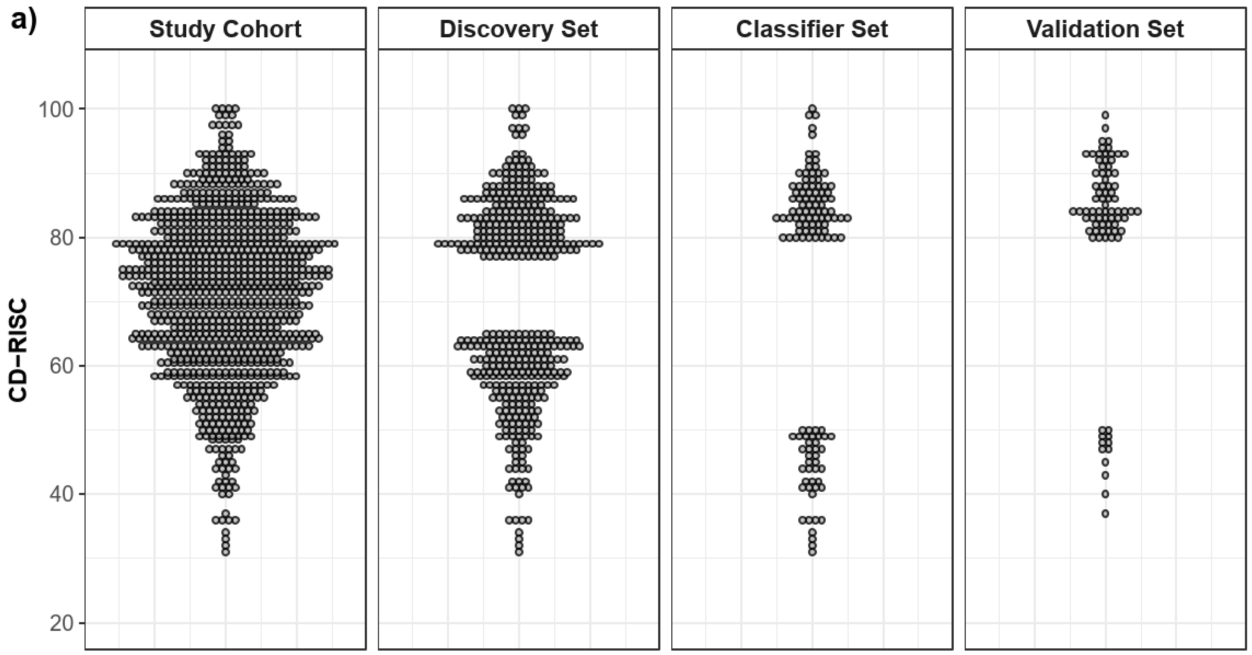

b)

Characteristic	Study Cohort Overall N = 934	Study Cohort High N = 617	Study Cohort Low N = 317	Discovery Set Overall N = 423	Discovery Set High N = 210	Discovery Set Low N = 213	Classifier Set Overall N = 123	Classifier Set High N = 82	Classifier Set Low N = 41	Validation Set Overall N = 80	Validation Set High N = 68	Validation Set Low N = 12
CD-RISC												
Mean	70.9	78.1	57.0	69.8	83.6	56.2	71.8	85.8	43.6	80.6	86.7	46.1
SD	12.5	7.7	7.2	15.1	5.0	7.6	20.6	4.7	5.6	15.4	5.0	4.1
Median	72.0	77.0	59.0	65.0	83.0	58.0	83.0	85.0	45.0	84.0	85.5	47.5
Min	31.0	66.0	31.0	31.0	77.0	31.0	31.0	80.0	31.0	37.0	80.0	37.0
Max	100.0	100.0	65.0	100.0	100.0	65.0	100.0	100.0	50.0	99.0	99.0	50.0

Figure S1: Distribution of CD-RISC scores in the study cohort and sample sets. a) Dot plot illustrating the distribution of CD-RISC scores with each dot representing an individual sample. **b)** Summary statistics of study cohort and sample sets depicted in panel a).

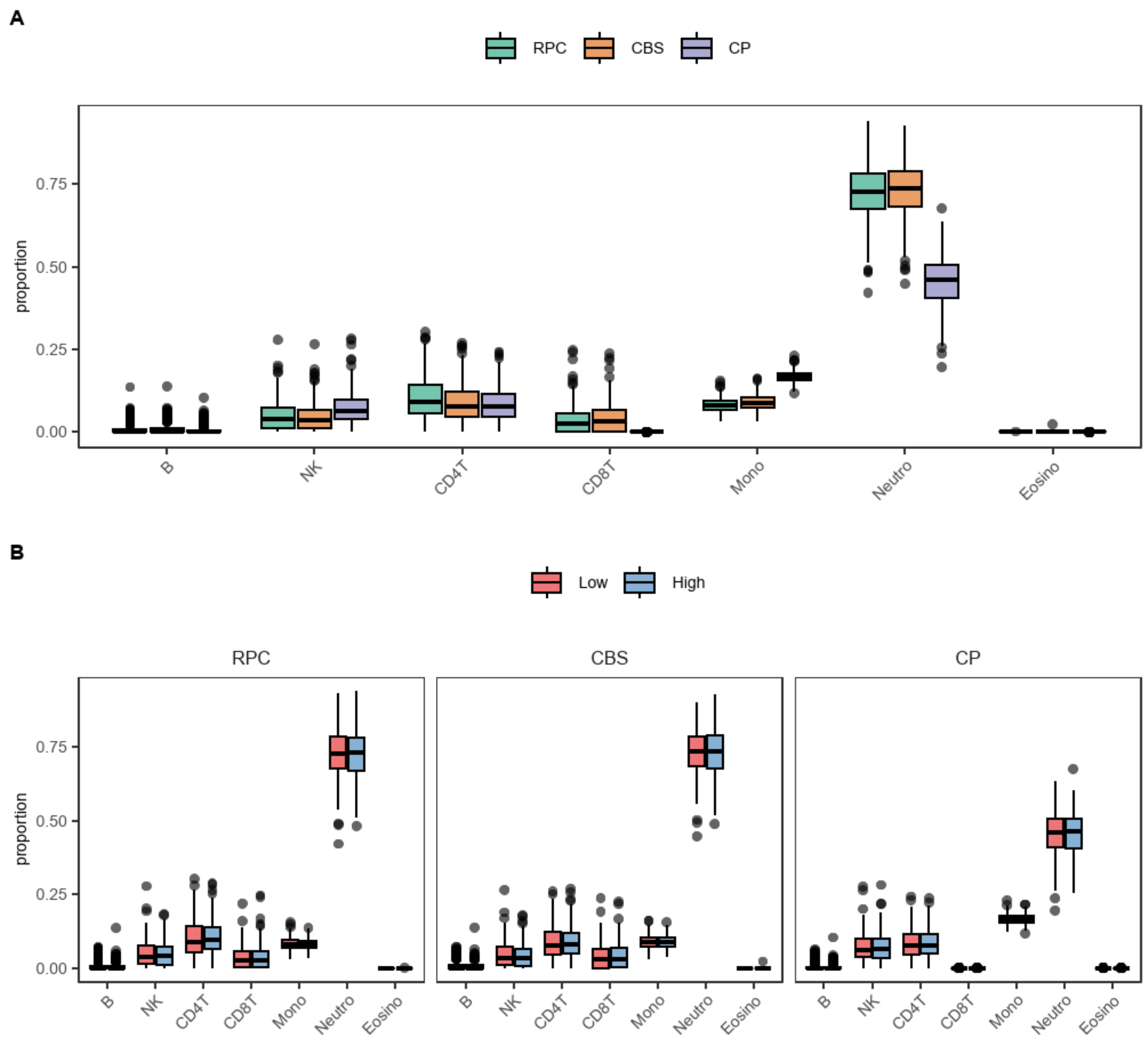

Figure S2: Cell type estimates. A) Comparison of proportions of B-cells (B), natural killer cells (NK), CD4-positive T-cells (CD4T), CD8-positive T-cells (CD8T), Monocytes (Mono), Neutrophils (Neutro), and Eosinophils (Eosino) estimated by Robust Partial Correlations (RPC), Cibersort (CBS) and Constrained Projection (CP). B) Comparison of the cell type proportions split by low (red) and high (blue) resilient sample group.

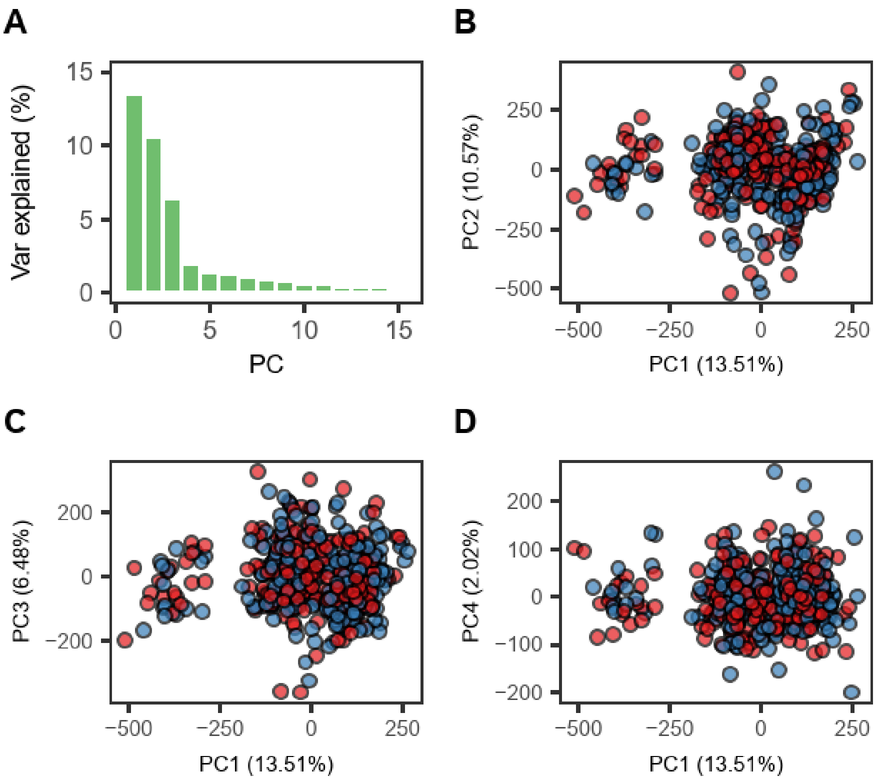

Figure S3: Principal Component Analysis of methylation profiles in the Discovery set. A) Percentage of variance explained by the first 15 components. B – D) PCA scores plots of PC1 vs. PC2, PC3 and PC4, respectively, with samples color-coded by low (red) and high (blue) resilience status.

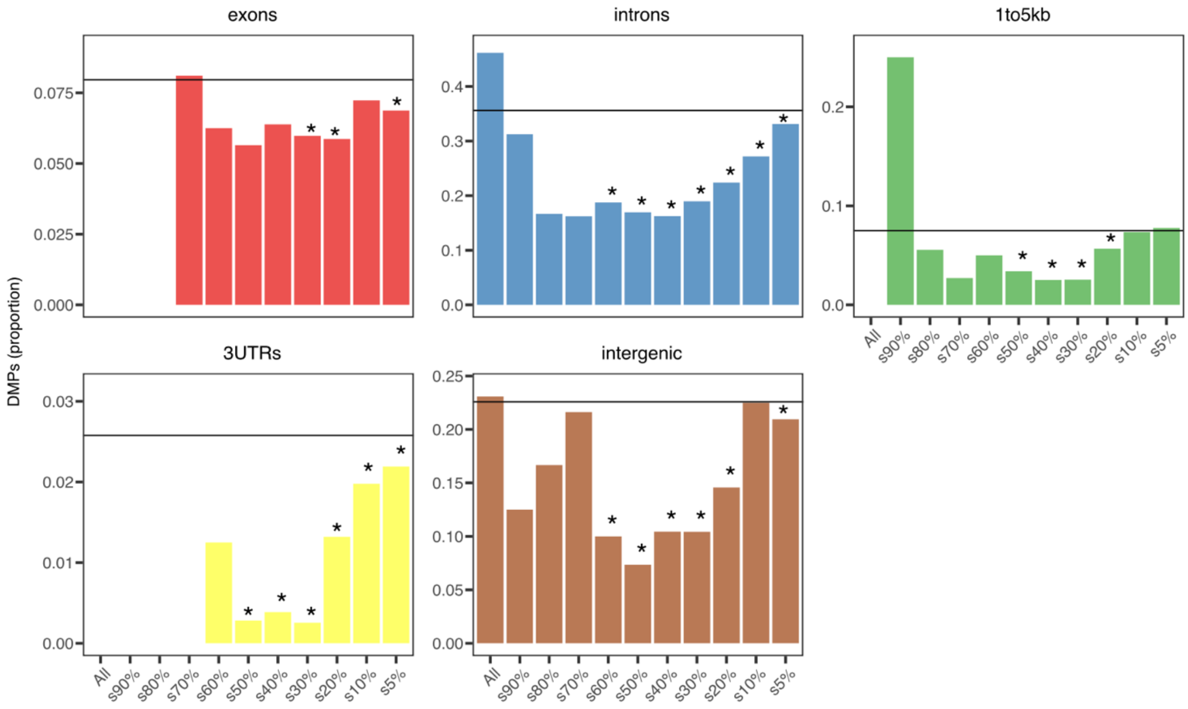

Figure S4. Proportions of DMPs overlapping with functional genomic regions. Horizontal reference lines correspond to proportions of all CpG, sites passing QC, found in the methylation array and falling in the respective functional regions. *) proportions significantly increased/decreased ($p < 0.05$).

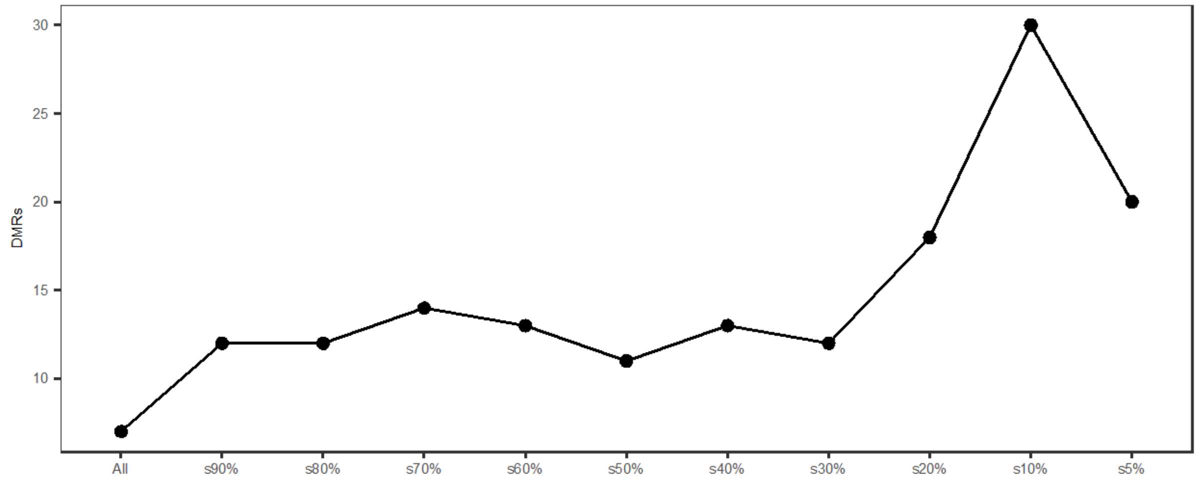

Figure S5: DMRs detected in subsets. Number of regions identified as differentially methylated regions (DMRs) between high- and low-resilient samples when comparing all samples in the Discovery set and across its ten subsets.

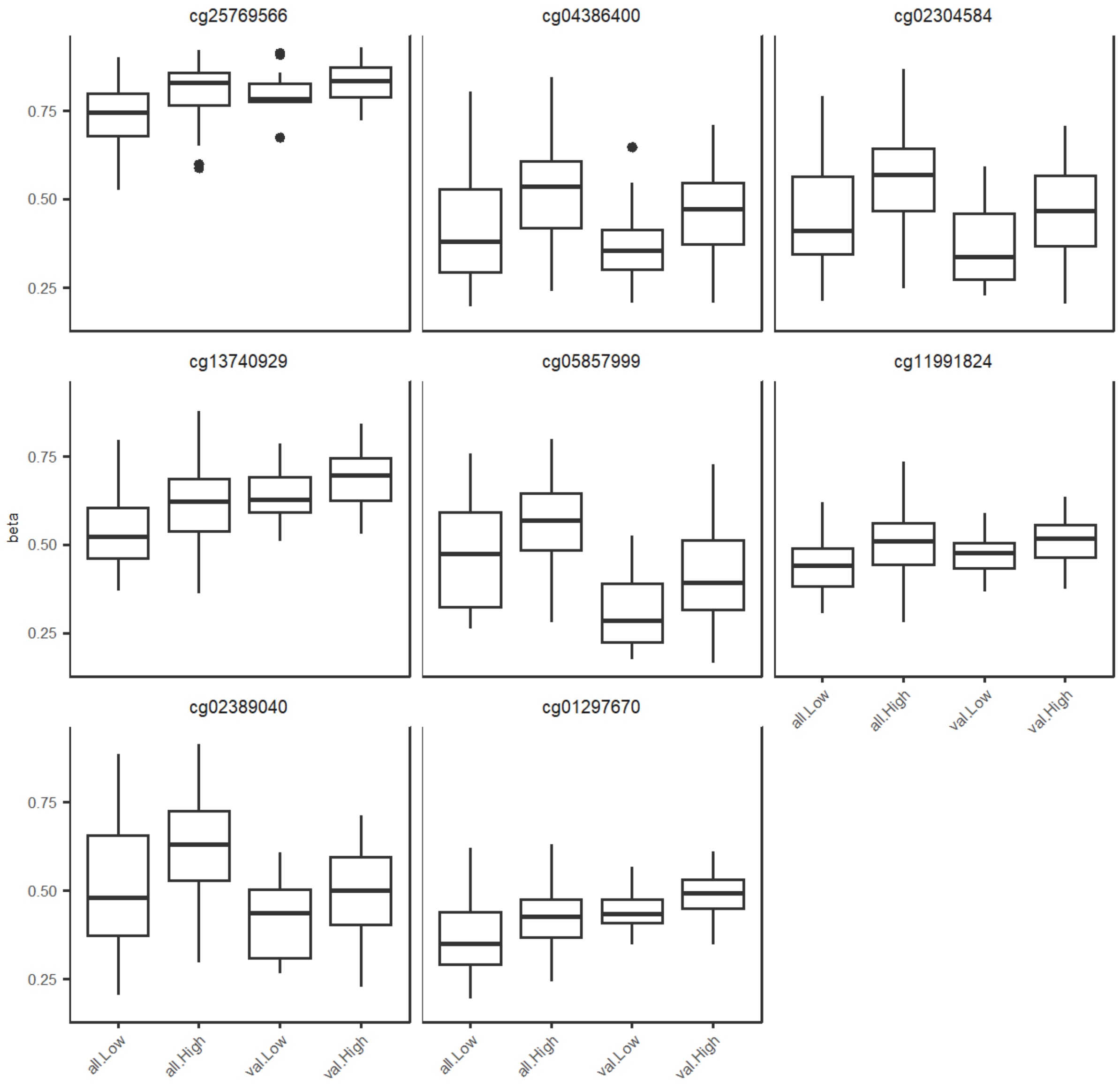

Figure S6: Distribution of methylation levels (beta values) at the CpG sites used to build the Random Forrest classifier. all.Low = low resilient samples in the classifier set, all.High = high resilient samples in the classifier set, val.Low = low resilient samples in the validation set, val.High = high resilient samples in the validation

Table S1: Association of clinical variables with the first five principal components

		PC 1		PC 2		PC 3		PC 4		PC 5	
	**df**[1]	**statistics**[2]	***P***[3]	**statistics**	***P***	**statistics**	***P***	**statistics**	***P***	**statistics**	***P***
**CD-RISC**	421	1.41	0.16	0.15	0.88	-0.67	0.5	1.33	0.19	-1.35	0.18
**Group**	1	0.52	0.47	0.42	0.51	0.17	0.68	3.89	0.05	1.13	0.29
**Age**	420	-1.87	0.06	-1.61	0.11	4.67	0	-4.7	0	-1.93	0.05
**Menstrual status**	1	1.08	0.3	1.08	0.3	7.5	0.01	7.2	0.01	0.16	0.69
**Detection mode**	1	0.82	0.36	0.12	0.73	0.51	0.47	0.66	0.42	3.57	0.06
**Stage**	4	2.45	0.65	1.75	0.78	3.8	0.43	0.78	0.94	0.58	0.97
**Histology**	3	4.13	0.25	2.57	0.46	2.29	0.51	1.65	0.65	2.74	0.43
**ER status**	1	0.06	0.81	0.48	0.49	1.4	0.24	0.68	0.41	1.66	0.2
**PR status**	1	0.13	0.72	0.9	0.34	0.33	0.56	0.72	0.39	1.38	0.24
**HER2 status**	1	0.01	0.91	1.4	0.24	0.82	0.37	0.27	0.6	0.77	0.38
**Smoking**	1	1.47	0.23	0.91	0.34	4.85	0.03	0.93	0.33	1.18	0.28

Association assessed based on Pearson's product moment correlation for continuous variables (CD-RISC, Age) and based on Kruskal-Wallis Rank Sum Test for remaining categorical variables. [1]degrees of freedom; [2]t statistics or Kruskal-Wallis rank sum statistics, [3]corresponding P val.
